# Supplementary material for: Association of VDR Polymorphisms with Muscle Mass Development in Elite Young Soccer Players: A Pilot Study
Source: Sports (Basel). 2024 Sep 13;12(9):253. doi: 10.3390/sports12090253 (PMC11436065; doi:10.3390/sports12090253)
Supplement: Supplementary file 1 [file sports-12-00253-s001.zip › table S2.pdf]

AMA				
**ApaI**	**BsmI**	**Frequency**	**Difference (95% CI)**	**p-value**
A	A	0.4164	0	---
C	G	0.3854	−0.94 (−4.73–2.86)	0.63
A	G	0.1654	−1.54 (−5.71-2.63)	0.47
C	A	0.0328	3.93 (−6.07–13.93)	0.44
**TMA**				
**ApaI**	**BsmI**	**Frequency**	**Difference (95% CI)**	**p-value**
A	A	0.4155	0	---
C	G	0.38466	−3.394 (-15.11 - 8.33)	0.57
A	G	0.1663	-4.36 (-17.32 - 8.61)	0.51
C	A	0.0336	11.28 (-20.5 - 43.05)	0.49

Table S2. Haplotype association with response with AMA and TMA.
