# Supplementary material for: Association of VDR Polymorphisms with Muscle Mass Development in Elite Young Soccer Players: A Pilot Study
Source: Sports (Basel). 2024 Sep 13;12(9):253. doi: 10.3390/sports12090253 (PMC11436065; doi:10.3390/sports12090253)
Supplement: Supplementary file 1 [file sports-12-00253-s001.zip › tableS1.pdf]

	età dec	sttt	peso (kg)	BMI	FFM Slaughter(Kg)	FFM Reilly (Kg)	AMA	CMA	TMA	playing position
1	14.99	176.4	63	20.25	55.42	57.14	46.42	76.1	141.42	midfielder
2	18.38	174.1	68	22.43	60.32	60.85	55.62	85.52	202.61	forward
3	18.02	175.7	68	22.03	59.57	61.31	45.81	94.38	185.98	defender
4	14.9	169	60.5	21.18	50.11	53.1	41.09	72.77	181.72	forward
5	15.02	172.4	67	22.54	55.5	59.16	44.84	90.13	190.41	midfielder
6	15.63	180	63	19.44	56.81	57.17	46.3	90.66	198.95	defender
7	16	175	66	21.55	59.03	59.71	52.77	92.67	191.75	forward
8	20.06	187.7	81	22.99	72.75	74.46	59.28	115.32	227.17	defender
9	14.77	166.8	60.5	21.75	52.78	54.51	53.42	92.67	183.57	midfielder
10	18.81	182.4	74	22.24	64.29	66.08	55.02	96.03	169.02	goalkeeper
11	16.57	162.5	60	22.72	53.23	54.51	56.4	89.97	192.96	forward
12	17.32	185	68	19.87	62.32	62.95	50.74	85.96	162.98	goalkeeper
13	15.19	168.1	69	24.42	56.9	60.72	60.35	95.72	204.33	goalkeeper
14	17.87	167.6	68	24.21	58.97	60.96	57.17	102.7	215.43	defender
15	15.02	193	87	23.36	69.5	76.32	52.66	101.16	196.69	goalkeeper
16	18.97	174.2	65	21.42	58.38	59.12	46.91	90.66	193.2	defender
17	14.58	169	65	22.76	56.47	57.37	51.72	73.81	209.45	forward
18	15.79	172.8	66	22.1	57.09	59.29	47.13	106.75	187.64	midfielder
19	15.62	172.8	66	22.1	58.06	59.53	47.02	105.66	195.45	forward
20	14.41	149.8	36	16.04	30.88	31.82	25.19	58.73	109.53	forward
21	16.99	184	75	22.15	64.88	68.3	66.77	98.9	216.49	defender
22	14.54	176.6	70	22.44	60.55	62.66	51.61	108.67	205.84	forward
23	16.92	195.3	92	24.12	67.41	77.88	63.53	98.35	221.76	goalkeeper
24	17.95	180.5	81	24.86	70.37	72.97	48.92	117.32	222.58	defender
25	14.1	171	56	19.15	50.91	50.97	40.72	72.62	145.08	midfielder
26	15.59	165.4	67	24.49	57.22	59.58	53.24	109.6	217.21	forward
27	18.42	174.4	72	23.67	60.17	63.78	53.95	99.14	219.95	forward
28	17.94	170	71	24.57	57.24	62.43	62.63	100.27	201.36	midfielder
29	16.96	177.8	66	20.88	60.73	60.78	54.84	102.62	231.56	midfielder
30	15.43	180.1	77	23.74	64.91	68.68	52.72	116.45	258.57	forward
31	17.33	169.7	63	21.88	54.73	55.94	43.02	91.28	214.37	midfielder
32	15.38	171.7	68	23.07	58.32	60.61	52.48	98.34	213.07	defender
33	17.81	165.4	71	25.95	60.64	63.06	59.35	100.03	212.5	forward
34	17.65	164.8	57	20.99	48.26	50.27	34.27	77.3	142.39	midfielder
35	18.81	174.2	66	21.75	58.06	60.28	55.56	100.38	204.67	midfielder
36	18.8	182	72	21.74	64.14	65.38	56.22	90.12	226.7	forward
37	16.23	166.5	56	20.2	50.5	51.37	42.54	96.98	168.17	midfielder
38	16.91	173	69	23.05	62.22	62.78	62.94	99.94	227.78	forward
39	16.78	176.5	72	23.11	60.7	64.66	53.19	99.06	216.73	defender
40	16.09	178.3	60	18.87	53.23	53.91	48.36	88.45	179.99	midfielder
41	17.28	169.2	64	22.36	56.07	57.45	43.66	93.76	209.2	defender
42	18.93	164	56	20.82	49.68	50.72	41.45	81.33	176.11	midfielder
43	18.06	175.5	73	23.7	59.39	63.63	40.68	98.5	194.89	midfielder
44	14.81	174.2	61	20.1	53.22	54.52	37.06	76.95	168.6	defender
45	15.07	176.7	62	19.86	55.91	56.13	38.94	85.44	182.37	forward
46	14.14	162.3	73	27.71	61	64.14	47.97	104.35	204.46	midfielder

47	14.72	174.2	63	20.76	53.57	55.46	44.14	89.9	191.97	defender
48	17.33	182.7	68	20.37	59.57	61.28	41.92	83.88	180.74	midfielder
49	18.93	182	82	24.76	70.93	72.49	65.33	96.59	214.03	defender
50	17.13	164.1	65	24.14	58.14	58.92	55.14	98.66	206.41	forward
51	17.04	178	66	20.83	59.28	60.36	50.22	94.38	218.74	forward
52	14.85	157.1	44.5	18.03	40.29	40.61	29.53	66.37	147.13	defender
53	18.9	173.7	74	24.53	60.75	65.09	57.75	101.32	202.27	forward
54	16.96	170.6	67	23.02	57.96	60.22	57.68	103.68	216.85	midfielder
55	16.99	179.2	64	19.93	56.54	58.03	47.02	101.72	187.3	defender
mean	16.65	173.73	66.99	22.13	57.92	60.03	50.02	93.82	196.15	
DS	1.55	8.18	9.01	2.06	6.90	7.74	8.64	12.11	26.60	

Table S1. Anthropometric characteristics of the sample.
